# The promising cancer treatment approach in cancer immunotherapy: dendritic cell-based vaccines

**DOI:** 10.3389/fimmu.2026.1902454

**Published:** 2026-07-24

**Authors:** Jingwen Wang, Xibiao Jia, Yaxin Zhang, Xiaoyu Wang, Yunhai Fu, Zhiyao He

**Affiliations:** 1 Department of Pharmacy, Cancer Center and State Key Laboratory of Biotherapy, West China Hospital, Sichuan University, Chengdu, China; 2 Key Laboratory of Birth Defects and Related Diseases of Women and Children, Ministry of Education, West China Second University Hospital, Sichuan University, Chengdu, Sichuan, China; 3 Key Laboratory of Drug-Targeting and Drug Delivery System of the Education Ministry and Sichuan Province, Sichuan Engineering Laboratory for Plant-Sourced Drug and Sichuan Research Center for Drug Precision Industrial Technology, West China School of Pharmacy, Sichuan University, Chengdu, China

**Keywords:** cancer, cancer vaccines, DC vaccines, dendritic cells, immunotherapy

## Abstract

Dendritic cells (DCs), as the most protent professional antigen-presenting cells (APCs), possess an exceptional capacity to initiate and modulate adaptive immune responses, rendering DC-based vaccines a highly promising approach in tumor immunotherapy. This review explores the biological properties of DCs, establishing a foundation for the rational development of DC vaccine. We further provide a comprehensive overview of current mainstream DC vaccine platforms, with analysis of three representative strategies: (i) DC vaccines loaded with tumor lysates; (ii) personalized neoantigen-pulsed DC vaccines; and (iii) DC-derived exosome vaccines, a rapidly emerging cell-free therapeutic modality. With respect to clinical translation, we summarize the outcomes of clinical trials conducted over the past five years, particularly targeting high-mortality malignancies. While the results are encouraging, the broad clinical application remains limited, posing several challenges, including functional heterogeneity of DCs and immunosuppressive tumor microenvironment, which need to be overcome. Combination strategies, such as DC vaccination paired with chemotherapy, radiotherapy or immune checkpoint blockade, are increasingly recognized as essential for enhancing therapeutic efficacy. With the sustainable progress in tumor immunobiology and advanced bioengineering in the future, the development of DC vaccines will be accelerated, offering novel therapeutic prospects for patients with refractory cancers.

## 1 Introduction

In the United States, cancer is the second-leading cause of death overall. According to the latest cancer statistics report, approximately 2,114,850 new cases and 626,140 cancer deaths are projected in 2026 (1). When zooming out to the worldwide public health landscape, a staggering estimate of 20 million cancer diagnoses worldwide and an unfortunate toll of approximately 9.7 million cancer deaths were witnessed in 2022 (2), underscoring the urgency of overcoming malignant tumors. Conventional therapeutic modalities, including surgery, radiotherapy, and chemotherapy, remain limited by high recurrence rates and narrow therapeutic windows, thereby motivating the pursuit of more effective strategies against cancers (3). Among emerging approaches, cancer vaccines have received widespread attention as a precise immunotherapeutic strategy to harness and amplify antitumor immunity.

**Figure 1:**
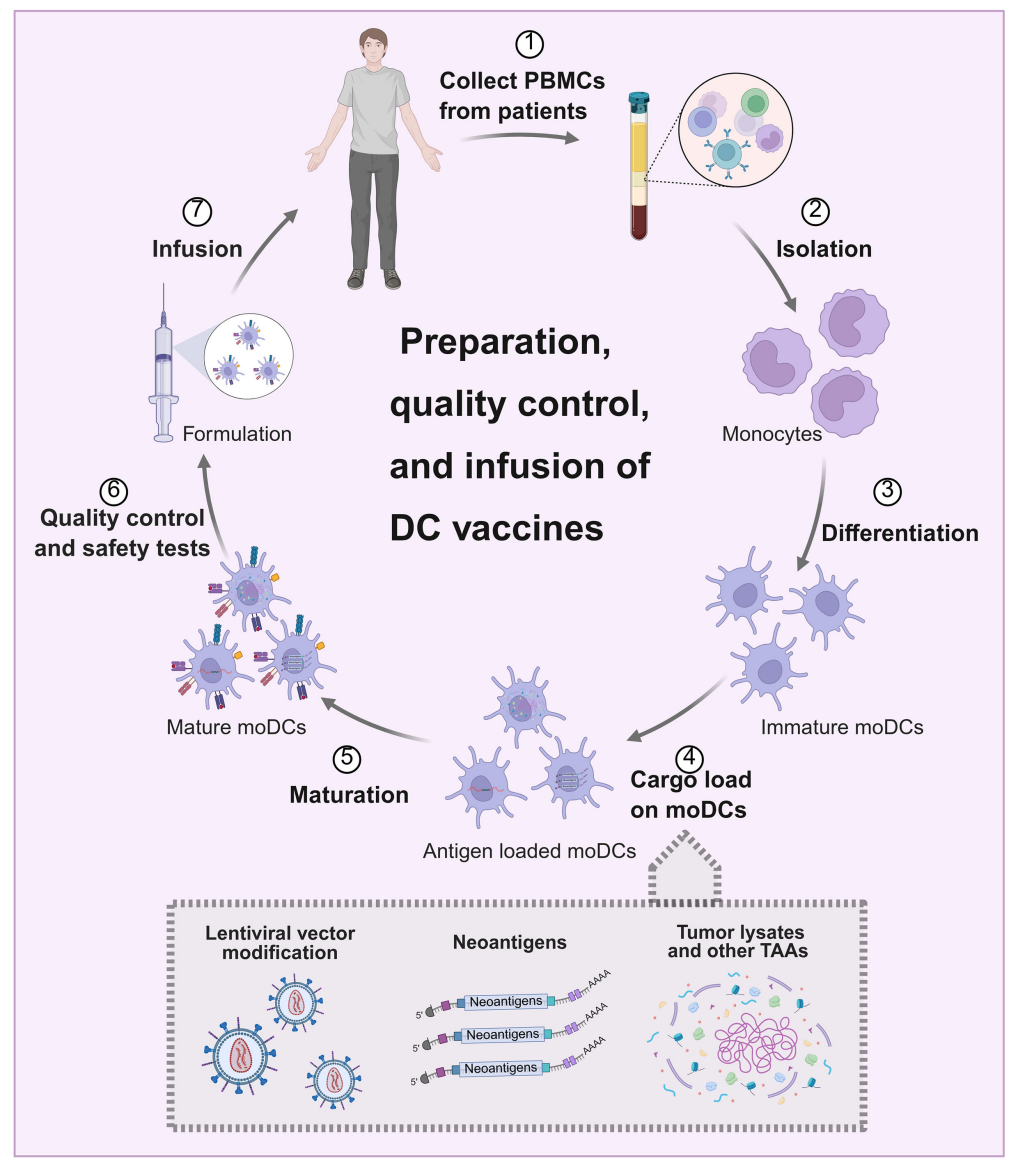
Preparation, quality control and infusion procedures of DC vaccines. Peripheral blood is collected from patients, and peripheral blood mononuclear cells (PBMCs) are isolated via density gradient centrifugation to obtain monocytes. Monocytes are subsequently differentiated into immature monocyte-derived DCs (moDCs) through stimulation with GM-CSF and IL-4. Antigens are then loaded onto immature moDCs using different loading methods (genetic engineering or direct antigen uptake), followed by maturation induction with TLR agonist and cytokines. After quality control and safety tests, the final vaccine formulation is reinfused into the patient.

The primary objective of cancer vaccines is to elicit antigen-specific adaptive immune responses targeting tumor-associated antigens (TAAs) and tumor-specific antigens (TSAs), thereby inhibiting tumor progression, inducing regression of established tumors and eradicating minimal residual lesions (4, 5). The efficacy of cancer vaccines hinges critically on efficient antigen presentation to naive T cells. Dendritic cells (DCs), as the most potent professional antigen-presenting cells (APCs), generally orchestrate cytokine-mediated inflammatory responses and prime antigen-specific T cell activation by recognizing and processing pathogen- and damage-associated molecular patterns (PAMPs/DAMPs) (6). Owing to their abilities to activate the immune response, DCs have great potential in cancer immunotherapy and DC-based vaccination is quickly and steadily advancing for the treatment of multiple types of cancer (7–9). And DC vaccines are manufactured in strict conformity with good manufacturing practice (GMP) guidelines (**Figure 1**), ensuring their reproducibility and safety. In 2010, Sipuleucel-T, the first autologous DC vaccine approved by the Food and Drug Administration (FDA), was authorized for the treatment of prostate cancer (10), validating the clinical feasibility of this platform. Recently, in 2024, the FDA granted orphan drug designation to Dubodencel (DOC1021) developed by Diakonos Oncology for malignant glioma.

Although DC vaccines demonstrate favorable safety, durable immunogenicity, and measurable clinical activity, their broader clinical translation remains hindered by several biological, technical, and logistical challenges. In this review, we introduce the functional heterogeneity of DC subsets in antitumor immunity and provide a comprehensive overview of current mainstream DC vaccine platforms. We further summarize the recent advances of DC vaccines, with emphasis on pivotal clinical trials that have yielded clinically meaningful outcomes. Finally, we critically analyze the current challenges like the impact of immunosuppressive microenvironment on vaccine efficacy. And combination therapy strategies are proposed to overcome these barriers.

## 2 Biological characteristics of DCs

**Figure 2:**
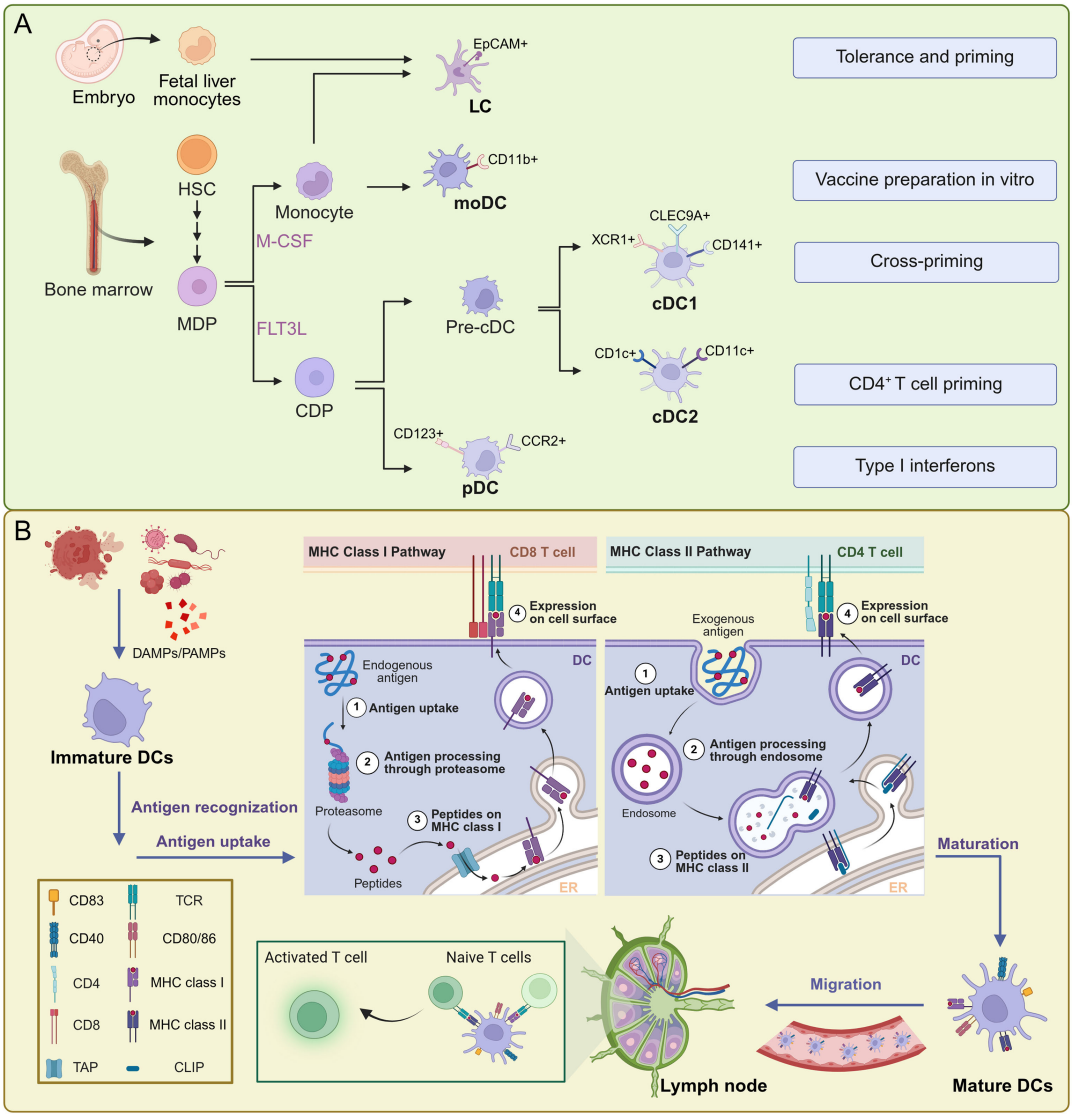
Subset classification and activation mechanism of DCs. **(A)** Subset classification of DCs. CDCs and pDCs arise from a common DC progenitor (CDP), and require the transcription factor FMS-like tyrosine kinase 3 ligand (FLT3L) for development and expression (11). CDCs are further divided into cDC1s and cDC2s. CDC1s are mainly responsible for cross-presenting antigens to CD8^+^ T cells, while cDC2s mainly present antigens to CD4^+^ T cells (12). PDCs can secrete a large amount of type I interferons, exerting antiviral and immune effects. Like classical monocytes, moDCs rely on the cytokine macrophage colony-stimulating factor (M-CSF) for differentiation, which are widely used for preparation of DC vaccines *in vitro*. LCs originate from fetal liver monocytes and bone marrow that act for immune tolerance in the skin. In addition, each subset express characteristic surface markers, reflecting the diversity and specificity of the DC lineage. **(B)** Activation mechanism of DCs. DCs recognize pathogen associated molecular patterns (PAMPs) or damage associated molecular patterns (DAMPs) through surface pattern recognition receptors (PRRs). Antigens uptake occurs through phagocytosis, endocytosis, or receptor-mediated endocytosis, enabling efficient internalization, processing and presentation of antigens. Exogenous antigens are degraded into short peptides within lysosomes, which bind to MHC class II molecules and are transported to the surface of DCs. In contrast, endogenous antigens are processed by proteasomes in the cytoplasm and loaded onto MHC class I molecules prior to surface presentation (14). Antigen recognition is accompanied by the activation of PRR mediated signaling pathways, which drive the upregulation of co-stimulatory molecules (CD80/CD86, CD40), inducing DC maturation and activation. Subsequently, DCs migrate to lymph nodes to promote the activation and proliferation of naive T cells, ultimately initiating the antigen-specific immune responses. ER, Endoplasmic reticulum; TAP, Transporter associated with antigen processing; TCR, T-cell receptor; CLIP, Class II-associated invariant chain peptide.

DCs, originating from hematopoietic stem cells (HSCs), are professional APCs that play a key role in initiating and shaping adaptive immune responses (**Figure 2A**) (13). They initiate the immune response by capturing antigens in peripheral tissues, processing into immunogenic peptides and presenting via major histocompatibility complex (MHC) molecules to naive T cells in secondary lymphoid organs (**Figure 2B**) (15). Based on ontogeny, which often correlates with transcription, phenotype, and function, DCs are classified into four principal subsets: conventional DCs (cDCs), monocyte-derived DCs (moDCs), plasmacytoid DCs (pDCs), and Langerhans cells (LCs) (11, 16).

CDCs represent the predominant APCs orchestrating adaptive immune responses. Based on distinct ontogeny, transcriptional programs and functional specialization, cDCs are broadly categorized into two major subsets: type 1 cDCs (cDC1s) and type 2 cDCs (cDC2s) subsets (17, 18). Typically, cDC1s cross-present exogenous antigens to naive CD8^+^ T cells via the MHC class I pathway (19, 20), thereby regulating cytotoxic T lymphocyte (CTL) responses against intracellular pathogens and TAAs derived from necrotic and apoptotic tumor cells (21). Beyond cross-presentation, cDC1s actively transport TAAs to tumor-draining lymph nodes (TdLNs), secrete immunostimulatory cytokines that regulate tumor-driven immunosuppression, and shape chemokine gradients to promote CTLs infiltration into the tumor microenvironment (TME) (22). These characteristics highlight the pivotal role of cDC1s in next-generation DC-based cancer vaccines. Nevertheless, clinical translation remains hampered by the scarcity of GMP-compatible methods for isolating and generating sufficient numbers of functionally intact cDC1s.

In contrast, cDC2s primarily present peptide-MHC class II complexes to CD4 T cells, driving helper T cell differentiation and modulating immunity against extracellular pathogens (23). The role of cDC2s in cancer immunity remains less defined than that of cDC1s, probably attributing to substantial heterogeneity and functions, which may exert distinct effects on antitumor immune responses (24). Some studies have demonstrated that the effective priming of tumor-specific CD4^+^ T cells also rely on cDC1s in early antitumor responses, emphasizing the functional overlap and cooperation between DC subsets (25–27).

MoDCs constitute the most abundant DC subset in human peripheral blood and are commonly used for vaccine preparation (28). Under inflammatory conditions, circulating monocytes are recruited to TdLNs and differentiate into moDCs and subsequently migrate into the TME (29–31). Although tumor-associated moDCs possess capacity for antigen uptake and processing, they are less efficient in activating naive antigen-specific T cells (24, 32). MoDCs generated *ex vivo* have been tested extensively in vaccine inoculation studies, which show their ability to cross-prime T cells and produce antitumoral cytokines like interleukin-12 (IL-12) (22). However, their limited lymph node homing efficiency following injection likely influences vaccine potency and contributes to poor clinical outcomes (22, 33).

Plasmacytoid DCs (pDCs), predominantly residing in blood and secondary lymphoid organs (34), share a common lymphoid progenitor origin with cDCs, but pDCs exhibit distinct genetic profiles (35). Additionally, pDCs express relatively low levels of MHC class II and co-stimulatory molecules under steady-state conditions (36). During viral infection, they serve as the principal producers of type I interferons (IFNs), linking innate and adaptive immune responses and exerting indirect antitumor effects (37). While pDCs are capable of cross-presenting antigens to CD8^+^ T cells, their limited efficiency in antigen cross-presentation suggests a relatively minor contribution to antigen-presenting (38).

Langerhans cells (LCs) originate from fetal liver monocytes that colonize the skin during embryogenesis (35, 39). At steady state, LCs migrate to skin-draining lymph nodes (sLNs) (40), where they promote the differentiation of regulatory T cells (Tregs) and contribute to immune tolerance in the skin (41), exhibiting immune functions that are different from those of cDCs.

## 3 Preclinical evaluation of various DC vaccines *in vivo*

As an immunotherapeutic platform capable of efficient antigen presentation, DC vaccines have demonstrated promising therapeutic potential in the treatment of various malignant tumors, primarily through the precise activation of tumor-specific T cells. To date, three mainstream DC-based vaccination strategies have been developed for tumor immunotherapy, including tumor lysate-loaded DC vaccines, neoantigen-pulsed DC vaccines (Neo-DCVac) and DC-derived extracellular vesicles (DEVs). Each approach possesses distinct advantages and limitations (**Table 1**), and numerous preclinical studies have confirmed the safety and efficacy of them, thereby providing a robust foundation for their clinical translation.

**Table 1:** Comparison of three DC-based vaccination platforms.

Comparison item	Tumor lysate-pulsed DC vaccines	Neoantigen-pulsed DC vaccines	DC-derived extracellular vesicles
Antigen types	TAAs and TSAs	Neoantigens	Antigens preloaded into DCs
Core advantages	Low cost; reducing the chance of tumor escape (42)	Low risks of immune tolerance and autoimmune reactions (43)	More resistant to tumor-mediated immunosuppression (44, 45)
Primary limitations and risks	High risk of triggering autoimmune response in patients; difficult to purify and quantify (46, 47)	High cost; time-consuming; hard to precisely identify (48)	Lack of standardized methods for high-throughput isolation and purification (49, 50)

### 3.1 DC vaccines loaded with tumor lysates

Tumor lysates represent a rich and broadly representative antigen source for vaccine development. Antigens derived from whole-tumor cell lysates can be directly internalized, processed, and presented by APCs (47), enabling simultaneous targeting of diverse tumor antigens and triggering potent specific antitumor responses (51). To date, the majority of DC vaccines are prepared using moDCs. In immunotherapy studies, tumor lysates were typically loaded into moDCs to produce lysate-pulsed moDC vaccines, which have shown favorable safety and encouraging clinical efficacy (52). Li et al. (53) generated neoantigen-reactive T cells (NRT) following immunization with an oxidized whole-tumor cell lysates pulsed DC vaccine (OCDC) vaccine. In this preclinical study, the OCDC vaccine induced neoantigen-specific immune responses in murine lung cancer *in vivo*. Moreover, combining OCDC vaccination with adoptive transfer of NRT modulated the TME, thus suppressing tumor growth and enhancing antitumor efficacy.

### 3.2 Neo-DCVac

Neoantigens are epitope peptides uniquely derived from nonsynonymous somatic mutations in malignant tumor cells (54), exhibiting high individual specificity (55). Unlike TAAs, neoantigens constitute a class of TSAs and are expressed exclusively in tumor cells (56). And they possess enhanced immunogenicity and higher binding affinity for MHC molecules (57), rendering them promising targets in tumor immunotherapy now.

Zhang et al. (58) comparatively evaluated the immune activation and antitumor efficacy of neoantigen-pulsed DC vaccines versus neoantigen-adjuvant vaccines using candidate neoantigens identified from murine lung carcinoma models. The results showed that neoantigen-pulsed DC vaccines elicited significantly stronger antigen-specific lymphocyte responses and conferred superior antitumor effects relative to neoantigen-adjuvant vaccines. This study provided critical preclinical evidence supporting the selection of neoantigen delivery platforms in personalized cancer immunotherapy. Hepatocellular carcinoma (HCC) is frequently characterized by an immunologically “cold” state, marked by sparse infiltration of CTLs, abundant immunosuppressive cells and impaired antigen presentation (59). This accounts for the poor responses of most HCC patients to monotherapy with immune checkpoint inhibitors (ICIs) (60, 61). To convert the immunosuppressive “cold” state into an “hot” state and reprogram tumor-associated neutrophils to potentiate antitumor immunity, Wang et al. (62) designed an acidic and photo-sensitive DC-based nanovaccine loaded with neoantigens. And this innovative platform effectively reshaped the TME by enhancing both TAA and TSA specific T cell responses, thereby potentiating the therapeutic efficacy of immunotherapy in HCC. Zhang et al. (63) developed a neoantigen-mRNA/DC vaccine using transmembrane peptides and murine colon cancer candidate neoantigens, which induced strong T cell immune responses, effectively preventing tumor growth. This study provided a feasible method for optimizing the production of DC vaccines and reducing costs.

### 3.3 DEVs

Extracellular vesicles (EVs) are nanoscale, lipid bilayer-enclosed particles secreted by all types of cells, which are broadly divided into four categories: exosomes, microvesicles, oncosomes, and apoptotic bodies (64, 65). EVs mediate intercellular communication by transferring bioactive molecules, such as nucleic acids, proteins and metabolites from donor to recipient cells through diverse mechanisms (66, 67). This characteristic provides a foundation for their potential applications as therapeutic agents and engineered drug delivery platforms. Natural EVs derived from tumor cells or immune cells have demonstrated intrinsic immunostimulatory properties that can trigger antitumor immunity, supporting their development as cancer vaccines (67). DEVs have the ability to overcome tumor-induced immunosuppression via direct and indirect methods (47, 68, 69). As a promising mode in cell-free therapeutics, DEVs are increasingly leveraged in the development of cancer vaccines (70).

The phospholipid bilayer of DEVs endows them with inherent structural stability, effectively protecting encapsulated contents from enzyme degradation and extracellular clearance (67), making it well-suited as versatile carriers for therapeutic biomolecules. Parisa et al. (71) evaluated the *in vitro* efficacy of DEVs loaded VEGF-A siRNA and doxorubicin as a multimodal drug against glioma. Their findings demonstrated that the engineered DEVs suppressed tumor proliferation, invasion, migration and angiogenesis while activating potent antitumor immune responses. DC-derived exosomes (DEXs) are a type of DEVs that can migrate to tumor sites or spleen and present antigens to CD4^+^ and CD8^+^ T cells, thereby inducing antigen-specific T cell responses (14). An aberrantly transcribed exon-intron*_antisense_* chimeric RNA *ASTN2-PAPPA_antisense_* (*A-P_as_* chiRNA) was recently identified as being selectively expressed in esophageal carcinoma (72). Xiong et al. (73) engineered DCs to express the chimeric RNA and employed the transfected DCs to produce a neoantigen-based anticancer vaccine. The vaccine showed robust antitumor efficacy, significantly inhibiting tumor growth and prolonging survival in preclinical models. And the study provided the first experimental evidence that cancer-specific transcription-derived chiRNAs can serve as immunogenic targets for producing cell-free cancer vaccines capable of eliciting CD8^+^ T cell-mediated anticancer immunity. Xu et al. (74) developed bispecific EVs (BsEVs) by genetically engineering EV-producing DCs to express the tumor homing molecule aCD19 single-chain fragment variable (scFv) and the immune regulatory molecule PD1. The engineered BsEVs simultaneously targeted tumor antigens and blocked PD1-mediated immune inhibition. The study revealed that BsEVs effectively primed antigen-specific CD8^+^ T cell responses and remodeled the TME, suggesting that this dual-targeting strategy may solve key limitations of current therapeutic EVs in tumor-targeted immunotherapy, especially for solid malignancies. Given that low migration level of DCs influenced cancer immune surveillance, Shang et al. (75) designed a nanovaccine comprising cationic nanoparticles loaded with immunogenic microvesicles derived from DCs and pulsed with patient-derived organoid or cancer cell lysates. This nanovaccine successfully converted immunologically “cold” tumors into “hot” ones, enhanced the recruitment and maturation of migratory DCs, activated both T cells and natural killer (NK) cells, and improved survival in orthotopic models of pancreatic ductal adenocarcinoma (PDAC) and lung cancer. Photodynamic therapy (PDT) could induce the immunogenic cell death (ICD), providing an ideal source of tumor antigens and danger signals for DC uptake. Leveraging this property, Wang et al. (76) developed an antigen-loaded exosomes (PDT-Dexs) derived from DCs pulsed with PDT-treated glioma cells. The results showed that PDT-Dexs promoted the activation of T cells *in vitro*, and when combined with PDT *in vivo*, they synergistically improved antitumor immunity. These findings highlighted the potential of PDT-Dexs as an exosome-based vaccine for patients with glioma.

## 4 Clinical trial results on DC vaccines

In light of the invasiveness and high recurrence rates of solid tumors, the development of DC vaccines has increasingly focused on rational and target-driven approaches. In contrast, within hematological malignancies particularly virus-associated diseases, the targeting specificity of DC vaccines confers a distinct therapeutic advantage. These cross-tumor studies not only encompass high-incidence tumor types with urgent clinical needs, but also systematically verify the adaptability and therapeutic efficacy of DC vaccines across different cancers through personalized antigen design and combination therapy strategies, establishing a diversified foundation for subsequent precise clinical translation. To date, a total of five DC vaccines has been approved globally (**Table 2**) and multiple clinical trials have reported exciting outcomes in recent years. Additionally, there are also several clinical trials ongoing (**Table 3**).

**Table 2:** Approved DC vaccines.

Product	Indication	Region	Time	Ref.
Hybricell	Advanced melanoma or renal cell carcinoma	Brazil	2005	(77)
CreaVaxRCC	Metastatic renal cell carcinoma	Korea	2007	(78)
DCVax-Brain	Brain cancer	Switzerland	2007	(79)
Provenge	Prostate cancer	America	2010	(10, 80)
APCEDEN	Refractory solid malignancies	India	2017	(81)

**Table 3:** Ongoing DC vaccine trials.

Vaccine/trial	Cancer type	Platform	Phase/status	NCT number
FRalphaDC	Ovarian cancer	Peptide-loaded vaccine	Phase I/II	NCT05920798
NeoDC-Vac	Esophageal squamous cell carcinoma	Neoantigen-loaded vaccine	Phase II	NCT06675201
CCL21-gene modified DC vaccine	Non-small cell lung cancer	Autologous DC-adenovirus CCL21 vaccine	Phase I	NCT03546361
YS247	Metastatic prostate cancer	Neoantigen-pulsed DC vaccine	Phase I	NCT07583160
AdHER2DC	Advanced or metastatic endometrial cancer	HER2 targeting autologous DC vaccine	Phase I/II	NCT06253494
Neo-DCvac	Advanced lung cancer resistant to ICIs	Neoantigen-loaded DC vaccine	Phase I	NCT06329908
CombiCoR-Vax	Metastatic colorectal cancer	Autologous DC vaccine	Phase II	NCT06522919
Prostate cancer vaccines	Prostate cancer	Tumor lysates-pulsed DC vaccine	Phase I/II	NCT07068555
MATCHPOINT	Medulloblastoma	Tumor RNA-pulsed DC vaccine	Phase I	NCT06514898

### 4.1 Hematological malignancies

Hematological malignancies constitute a large and heterogenous group of neoplasms affecting the hematopoietic and outcomes, including myelodysplastic syndromes (MDS), diffuse large B-cell lymphoma (DLBCL) and acute lymphoblastic leukemia (ALL) (82). These cancers are characterized by dysregulated proliferation of hematopoietic stem cells or the progenitor cells, leading to normal hematopoietic dysfunction. And many hematologic cancers remain incurable, frequently relapsing or progressing to refractory diseases (83), appealing for innovative strategies to enhance treatment efficacy and improve prognosis. DC vaccines, serving as an important modality of cancer immunotherapy, have shown excellent safety profiles, consistent immunogenicity and modest clinical efficacy (84).

FDC101, an autologous mature DC vaccine loaded with transcribed RNA (ivt-RNA) encoding Wilms tumor 1 (WT1) and Preferentially expressed Antigen in Melanoma (PRAMEF), was tested in a phase I/II trial enrolling twenty patients with acute myeloid leukemia (AML). They had achieved complete remission (CR) following induction chemotherapy and were deemed ineligible for allogeneic stem cell transplantation (ASCT) (85). Eleven of them remained in CR, and overall survival (OS) rate at five years was 75%, with 70% of patients aged ≥ 60 years surviving long-term. Maintenance therapy with FDC101 was well tolerated in AML patients in CR and yielded encouraging 5-year survival outcomes (NCT02405338). A phase I clinical study found that vaccination with autologous LCs electroporated with triple antigen-encoding mRNA (CT7, MAGE-A3, and WT1) following ASCT was safe and feasible in patients with multiple myeloma (MM) (NCT01995708). The results suggested that post-transplant vaccination represented a promising immunotherapeutic strategy to enhance antitumor responses after ASCT in MM (86). Additionally, a DC-based vaccine targeting EPS8 and WT1 antigens had shown clinical efficacy in prolonging CD19-targeting chimeric antigen receptor (CAR)-T cell persistence and improving outcomes in adults with refractory or relapsed B-cell acute lymphoblastic leukemia (B-ALL) (87). The trial yielded markedly improved results, with a 100% CR rate and a median leukemia-free survival of 489 days. CAR-T cell persistence was enhanced with CAR-T cell levels peaking within 7 days post-infusion and persisting for a median of 336 days (NCT03291444). For Epstein-Barr virus-associated lymphoproliferative diseases (EBV-LPDs), the KSD-101 vaccine, which loaded EBV-associated antigens to accurately activate specific T cells to clear virus-infected cells, has demonstrated favorable safety and efficacy (88). The best objective response rate (ORR) was 88.9% (8/9), and all 9 patients achieved CR at a median follow-up of 54.1 weeks (NCT05635591). The results established KSD-101 as a novel immunotherapeutic option for patients with relapsed and refractory hematological malignancies. These clinical studies provided a practical pathway for individualized treatment of hematological cancers through rational antigen selection and optimized combination strategies.

### 4.2 Glioblastoma

Glioblastoma (GBM) is a highly aggressive and lethal brain malignancy primarily affecting adults between the ages of 45 and 70. It is characterized by rapid proliferation and invasiveness, which often lead to poor prognosis despite standard of care (SOC) treatment (89). To date, clinical outcomes for GBM patients have shown minimal improvement and the incidence of GBM appears to be increasing for unclear reasons, underscoring an urgent need for new and better therapeutic strategies.

The autologous tumor lysate-loaded DC vaccination (DCVax-L) was a personalized immunotherapy that had yielded exciting results in a phase III, nonrandomized controlled trial (90). The median OS for patients with newly diagnosed GBM receiving DCVax-L reached 19.3 months from randomization, higher than the 16.5 months observed in control group. Survival at 48 months had improved from 9.9% to 15.7%, and at 60 months had increased from 5.7% to 13.0%. In patients with recurrent GBM, DCVax-L also demonstrated substantial clinical benefit that the median OS was extended from 7.8 months to 13.2 months, and survival at 24 and 30 months also rose from 9.6% and 5.1% to 20.7% and 11.1%, respectively (NCT00045968). Another phase II study evaluated AV-GBM-1, a DC vaccine generated by incubating autologous DCs with a lysate of irradiated autologous tumor-initiating cells, and treatment with the vaccine was well-tolerated in newly diagnosed GBM patients (NCT03400917). However, the OS at 2 years of AV-GBM-1 was 27%, lower than the 35% reported in the recently published phase III DCVax-L study (91). Notably, in 2025, DOC1021, a next generation DC vaccine loaded with autologous tumor lysate and amplified tumor-derived mRNA had shown breakthrough progress in GBM treatment. As adjuvant therapy following completion of standard chemoradiation, DOC1021 had increased OS at 12-months to 88%, far exceeding the expected 60% of receiving SOC alone (NCT04552886). And it offered meaningful improvement in the poor prognosis of patients with GBM multiforme (92), bringing new hope to individuals with this brain cancer.

### 4.3 Lung cancer

In recent years, ICIs and targeted therapy for lung cancer have developed rapidly, but overall clinical efficacy is still unsatisfactory, and lung cancer treatment still faces urgent clinical need. Concerning the promising therapeutic effects of DC-based vaccine targeting neoantigens in certain malignant tumors, Ding et al. (93) conducted a clinical trial in patients with advanced lung cancer using a personalized neoantigen peptide-pulsed autologous DC vaccine. In this study, the objective response rate was 25%, and the median OS was 7.9 months, providing new evidence for neoantigen-directed vaccine therapy and opening novel therapeutic opportunities for lung cancer treatment (NCT02956551). Another research team reported results from a phase I clinical trial evaluating a DC vaccine targeting patient-specific neoantigens in patients with resected non-small cell lung cancer (NSCLC) (94). The vaccination induced long-lived neoantigen-specific T cells with a high degree of differentiation heterogeneity, and durable T cell responses were observed in five out of six vaccinated patients for up to 19 months post vaccination. The data proved that the safety and feasibility of the vaccine (NCT04078269).

### 4.4 Pancreatic cancer

A novel WT1 peptide-pulsed DC (WT1-DC) vaccine combined with multiagent chemotherapy, had been shown to modulate the TME and facilitate conversion to surgery in pancreatic cancer (95). In a phase I trial of DC-based immunotherapy, an estimated 2-year recurrence-free survival (RFS) rate of 64% was achieved following pancreatectomy, SOC treatment and adjuvant DC-based immunotherapy. And vaccination led to increased percentages of activated peripheral blood CD4^+^ T cells and detection of treatment-induced vaccine-specific immune responses (96). This means using DC therapy as an adjuvant may restore DC-mediated immunity in patients with resected PDAC.

### 4.5 Hepatocellular carcinoma

HCC is usually diagnosed at an advanced stage, and local recurrence following potentially curative resection or ablation remains an inevitable clinical challenge (97). A study published in 2024 evaluated the efficacy of a personalized neoantigen-based mRNA loaded DC vaccine (LK101) administered in combination with conventional ablation in patients with HCC (98). Patients were followed for a median duration of 48.4 months in the vaccination arm, longer than 38.8 months in the control arm. The 1-year and 2-year recurrence rates were 18.2% versus 33.3%, and 36.4% versus 51.4%, respectively, in the vaccination arm compared with the control arm (NCT03674073). These findings implied that in combination with standard ablation therapy, a neoantigen-based mRNA loaded DC vaccine represents a promising immunotherapy strategy for HCC.

## 5 Barriers to translational application of DC vaccines from preclinical research to clinical practice

### 5.1 Immunosuppressive effect of the TME

**Figure 3:**
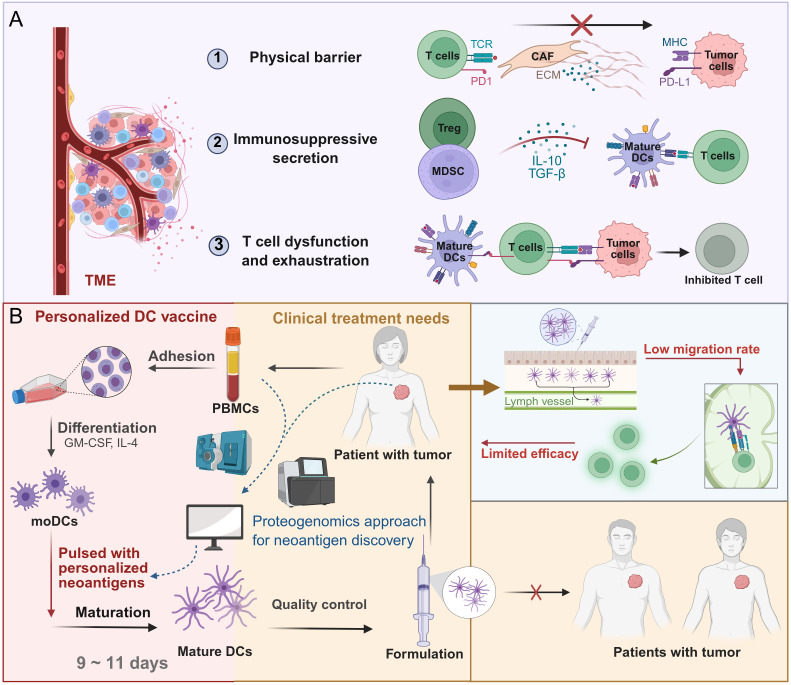
Challenges in the clinical translation of DC vaccines. **(A)** Immunosuppressive effects of the TME. The TME is a complex ecosystem composed of tumor cells, immune cells, cancer-associated fibroblasts (CAFs) and extracellular matrix (ECM). It can inhibit T cell function through multiple mechanisms, including the physical barrier formed by ECM hindering T-cell infiltration, immune checkpoint molecules (such as PD-L1/PD-1) blocking activation signals. Tregs and myeloid-derived suppressor cells (MDSCs) in the matrix secrete cytokines like IL-10 and TGF-β to inhibit the function of effector T cells, forming an immunosuppressive microenvironment (8). **(B)** Barriers to clinical translation. The production cycle of DC vaccines according to GMP standards is relatively long, and it takes 9–11 days to produce moDCs alone. The subsequent procedures, including quality control and safety assessments of the vaccine products, prolong the production cycle as well. The current advances in personalized neoantigen-based vaccines have attracted more attention, but the identification and validation of patient-specific tumor antigens remain costly. Moreover, the individualized vaccines exhibit limited cross-patient applicability. And the DCs reinfused into the patients may result in poor vaccine efficacy due to their low migration efficiency to lymph node.

The immunosuppressive network within the TME represents the primary barrier to the efficacy of DC vaccines (**Figure 3A**). Even if DC vaccines successfully prime naive T cells, the effector T cells infiltrating the TME often become functionally impaired due to aberrant activation of the programmed death-1 (PD-1)/programmed death-ligand 1 (PD-L1) pathway, leading to attenuated vaccine efficacy (99, 100). Solid tumors including GBM and pancreatic cancer are characterized by substantial infiltration of Tregs and myeloid-derived suppressor cells (MDSCs) within the TME. These immunosuppressive populations inhibit DC maturation and antigen presentation, down-regulate expression of critical co-stimulatory molecules (CD80, CD86 and CD40), and impair MHC molecule presentation by secreting various inhibitory cytokines like IL-10 and transforming growth factor-β (TGF-β) (101–104). In patients with advanced-stage disease, dominant immunosuppressive mechanisms result in diminished responses to DC vaccines and T cell activation, contributing to decreased clinical efficacy in those with metastatic disease (105, 106). For instance, in a single-agent trial of DC vaccine for pancreatic cancer without combination therapy, the 2-year RFS rate was less than 40%, significantly lower than the 64% observed in the concomitant therapy (96) (NCT04927780).

### 5.2 Limitations of DC function and antigen delivery in preclinical studies

An ideal DC vaccine should be fully matured *in vitro* to ensure robust and substantial T-cell activation. However, current protocols for *in vitro* induction and maturation frequently fail to replicate the complexity of the native TME *in vivo*, resulting in DCs with incomplete maturation or phenotypic instability (107). Immature DCs not only exhibit weak antigen-presenting capability, but may also promote T-cell tolerance or expansion of Tregs, thereby suppressing antitumor immunity (108, 109). Moreover, DCs are not a single colony, and functional heterogeneity exists across distinct DC subsets. Autologous peripheral blood moDCs are commonly used to prepare DC vaccines. But they have different biological characteristics from natural cDC1 and exhibit significant inter-individual variability, limited *in vitro* expansion efficiency, and inconsistent maturation outcomes (110), potentially weakening the CTL response. In patients with advanced tumors, the function of peripheral blood monocyte is impaired, and the differentiated DCs often show inadequate expression of costimulatory molecules (CD80 and CD86), rendering them suboptimal for effective T-cell activation (33).

The antigens loaded in DC vaccine should be tumor-specific, highly immunogenic, and broadly express across tumor cells. However, DC vaccines usually utilize well-characterized traditional TAAs now, which suffer from heterogeneous expression and carry risks of antigen loss or immune escape. In contrast, neoantigens, arising from somatic mutations, exhibit superior tumor specificity and have emerged as key components of personalized cancer immunotherapy. Nevertheless, the screening, validation and preparation procedures for individualized neoantigens are complex and costly, making large-scale promotion difficult (111) (**Figure 3B**). The utility of neoantigen-based vaccines is also limited by the diversity in mutational burden and spectrum across different tumor types (57). And personalized neoantigens have high patient specificity, making them unsuitable for a large number of patients (112).

Besides, inadequate antigen loading efficiency and antigen presentation quality can also undermine the overall efficacy of DC-based cancer vaccines. Common antigen loading strategies present distinct limitations. For instance, Peptide pulsing depends on the known HLA types, resulting in limited applicable population (113). Tumor lysates offer a broad antigen spectrum but contains non-specific components, which may induce autoimmune responses. Furthermore, the reinfused DC vaccine must effectively migrate to the lymph nodes to interact with naive T cells (8). However, due to the restricted migration efficiency and lack of effective *in vivo* targeting strategies, only a few DCs can successfully home to lymphoid tissues. And nanotechnology is likely to be an opportunity to tackle the challenges (114).

### 5.3 Practical obstacles in human clinical translation

Under GMP conditions, only one single-cell product can be processed at one manufacturing run, and moDCs generation requires 9 to 11 days (115) (**Figure 3B**). However, the preparation of DC vaccines involves a series of complicated processes, including monocyte isolation, *in vitro* culture, antigen loading, and quality control, which takes longer and renders the vaccines difficult to meet the urgent treatment needs of patients with advanced diseases. Because of the high degree of heterogeneity in tumors, the protocol for preparing individualized vaccines has poor reproducibility and poses significant obstacles for the development personalized neoantigen vaccines (116). Meanwhile, the preparation process and strict quality supervision requirements of personalized DC vaccine drive up the total cost, and it is hard to reduce costs due to limited mass production (117). Finally, the absence of standardized biomarkers, such as DC lymph node homing efficiency or T-cell receptor repertoire diversity, for evaluating immunological and clinical efficacy hampers the horizontal comparison of clinical research outcomes (118).

## 6 Approaches to enhance vaccine efficacy

Although most personalized therapeutic vaccines exhibit favorable safety with low toxicity, their clinical efficacy remains modest. Rational combination strategies, for example, co-administration with chemotherapy, radiotherapy, ICIs or adoptive cell therapies, represent a promising avenue to potentiate antitumor immunity and improve therapeutic outcomes (119).

**Figure 4:**
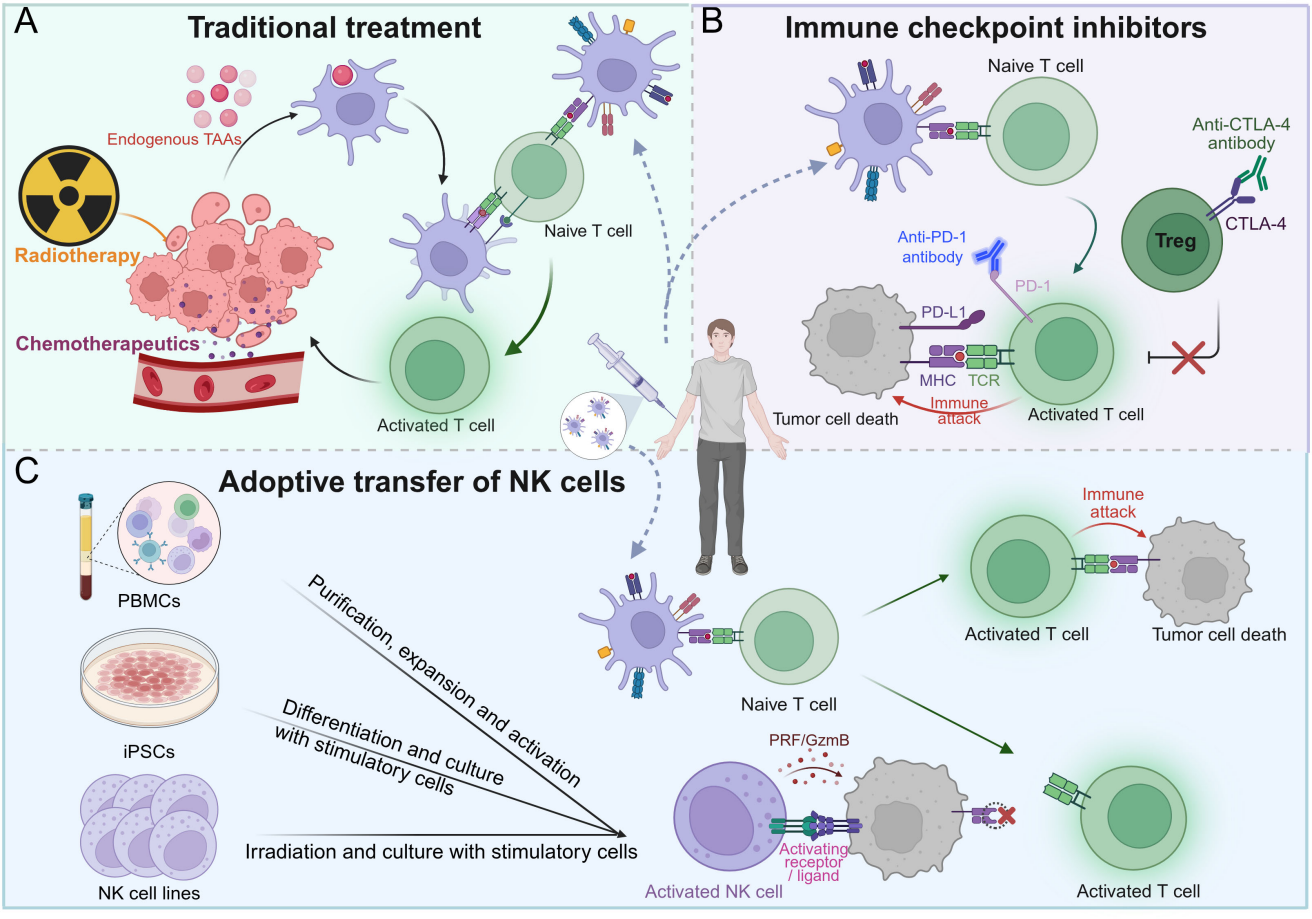
Combination therapy strategies to enhance the clinical efficacy of DC vaccines. **(A)** Combination with conventional therapies. Tumor irradiation and certain chemotherapeutic drugs can induce ICD, leading to the release of endogenous TAAs. These antigens bind to specific receptors on the DC surface, thereby increasing antigen uptake, processing, and DC maturation (28, 102). These DCs subsequently migrate to lymph node, where T cell priming occurs, compensating for the low lymph node homing capacity of reinfused DCs. **(B)** Combination with ICIs. ICIs can specifically block inhibitory immune checkpoint pathways. PD-1 inhibitors relieve tumor cells “immune escape” inhibition on T cells, while CTLA-4 inhibitors can selectively deplete Tregs within the TME, thereby relieving their suppression of effector T cells and further promoting T-cell activation. The combination of DC vaccine and ICIs yields a synergistic effect that can activate immunity and relieve inhibition (123). **(C)** Combination with adoptive transfer of NK cells. As the core effector cells of innate immunity, NK cells can directly kill tumor cells that downregulate or lack surface MHC I molecules, and can quickly initiate antitumor responses. DC vaccines can activate antigen-specific T cell response and further enhance NK cell function, synergistically combating tumors and reducing the risk of tumor immune escape.

The efficacy and clinical application of conventional tumor treatment modalities are often constrained by several inherent limitations. For instance, the therapeutic benefit of chemotherapy is undermined by chemoresistance, while the widespread application of radiotherapy is limited by the relatively low tolerance of healthy tissues to ionizing radiation (120). Nevertheless, both chemotherapy and radiotherapy can trigger ICD in tumor cells, thereby facilitating the uptake of TAAs by DCs and promoting DC maturation and activation. These immunomodulatory features suggest a likely favorable therapeutic effects from adjunct DC-based vaccination (**Figure 4A**) (121). A randomized phase II trial of an autologous active cellular immunotherapy consisting of DCs for ovarian cancer (DCVAC/OvCa), showed improved progression-free survival (PFS) in patients with ovarian cancer (NCT02107937). Among patients with stage III disease, median PFS was 21.4 months in group C consisting of patients who received chemotherapy alone, not reached in group B consisting of patients who received DCVAC/OvCa sequential to chemotherapy, and 20.3 months in group A consisting of patients who were treated with DCVAC/OvCa parallel to chemotherapy. A trend toward improved OS was also observed, with estimated OS at 4 years of 71%, 79% and 63% in group A, B and C, respectively (122). The results indicated that DCVAC/OvCa combined with chemotherapy appeared to induce a durable antitumor immune response and stabilize the disease, and prolonged survival in patients with ovarian cancer.

Since the FDA first approved the cytotoxic T-lymphocyte-associated antigen 4 (CTLA-4) antibody in 2011, ICIs have revolutionized cancer treatment over the past few years and established new treatment standards. However, the clinical efficacy of ICIs remains restricted in many patients due to primary or acquired resistance. And combining cancer vaccines with ICIs appears to be a rational and suitable option to potentiate synergistic treatment effects (**Figure 4B**) (124). The combination of a personalized neoantigen-loaded moDC (Neo-moDC) vaccine and nivolumab achieved complete and durable regression of gastric tumor for over 25 months in a patient with advanced metastatic gastric cancer (125). This study demonstrated that the combination therapy synergistically alleviated T-cell exhaustion and reshaped the TME by effector T cells in conjunction with PD-1 inhibitors. Multiple antigen-stimulating cell therapy-I (MASCT-I) represents the first application to combine DC vaccine and adoptive cellular therapy into a single treatment platform, thereby eliciting both active and passive immune response (126). Previous clinical studies showed that MASCT-I alone or in combination with apatinib conferred clinical benefit and exhibited safety profile in patients with advanced solid tumors like HCC (126, 127). In 2023, a clinical trial using MASCT-I plus camrelizumab and apatinib demonstrated encouraging efficacy in advanced bone and soft-tissue sarcoma (NCT04074564) (128). Another phase I trial reported that MASCT-I combined with camrelizumab achieved a 2-year PFS rate of 55.6% and a 48-month duration of response (DOR) rate of 71.8% in patients with metastatic urothelial carcinoma (NCT03034304) (129). These studies provide evidence that ICIs can potentiate the antitumor activity of DC vaccine, surpassing the efficacy observed with either DC vaccination or ICI monotherapy. Recently, Chen’s team conducted a randomized phase II clinical trial, to evaluate whether the combination of individualized Neo-DCVac and camrelizumab can improve OS in patients with unresectable locally advanced esophageal squamous cell carcinoma (ESCC) following definitive chemoradiotherapy (NCT06675201). Clinical outcomes were not yet available, but if effective, this strategy could offer a novel, personalized immunotherapeutic strategy for this population with limited post-treatment options (130). Moreover, immunomodulatory drugs (IMiDs) including lenalidomide and pomalidomide exhibited prominent synergistic effects when combined with DC vaccine in murine myeloma models. Lenalidomide plus PD-1 blockade treatment synergistically enhanced the efficacy of DC vaccine, while pomalidomide plus dexamethasone boosted the antitumor activity of DC vaccine by reshaping the TME, providing a preclinical rationale for developing a more advanced immunotherapeutic modality against multiple myeloma (131, 132).

NK cells are key effectors of the innate immune system, capable of eliminating target cells through integration of signals from activating and inhibitory receptors (133). The clinical application of NK cell-based therapies was initially pioneered in the context of hematopoietic stem cell transplants (HSCTs), guided by mechanistic insights into NK cell activation (134). Early successes of adoptive NK cell transfer in hematological cancers prompted the development of using the strategy against solid cancers. Combining DC vaccines with adoptive NK cell therapy offers a highly synergistic immunotherapeutic approach. The former activates antigen-specific T cells to mediate tumor cell lysis, while the latter eliminates cancer cells that escape the attack from T cell with the ability to non-specific killing (**Figure 4C**) (135). A clinical case reported that after receiving multiple circles of chemotherapy, followed by WT1-pulsed DC vaccination, adoptive NK cell therapy and nivolumab, the patient with bilateral ovarian cancer and multiple liver and lung metastases showed a disappearance of ascites and a remarkable regression of both primary and metastatic lesions throughout the body (136). This combined treatment strategy has demonstrated remarkable clinical efficacy and provides a feasible strategy for further enhancing the potency of DC vaccines. Beyond the NK cell-based therapies, generation of multipotent DCs with the addition of IL-15 represented another robust optimization approach. The resulting IL-15 DCs demonstrated strong cytolytic activity against myeloma cells, proposing a novel approach for DC-based immunotherapy (137).

## 7 Conclusion

Cancer is the second leading cause of death globally, and patients often face significant cardiovascular and psychological challenges (138), highlighting the necessity and urgency of cancer treatment. DC vaccines have demonstrated considerable therapeutic promise across diverse malignancies through the activation of the immune system, particularly by overcoming the limitations of single-agent efficacy in combination therapies. With significant advancements in high-throughput sequencing and antigen prediction technologies, the design of personalized cancer vaccines is becoming increasingly rational, and vaccine efficacy is expected to improve substantially. Furthermore, sophisticated bioinformatic algorithms and predictive modeling techniques are playing an increasingly pivotal role in accelerating the preclinical optimization and clinical translation of these vaccines. Beyond technological challenges in vaccine development, equal attention must be given to patient affordability and accessibility. High manufacturing costs may severely limit cost-effectiveness and hinder widespread clinical adoption following regulatory approval. Nevertheless, despite these challenges, substantial opportunities remain for innovation in cancer vaccine development, and their therapeutic potential in oncology is poised to be progressively realized in the years ahead.

## References

[1] Siegel RL Kratzer TB Wagle NS Sung H Jemal A . Cancer statistics, 2026. Ca-Cancer J Clin. (2026) 76:e70043. doi: 10.3322/caac.70043 41528114 PMC12798275

[2] Filho AM Laversanne M Ferlay J Colombet M Pineros M Znaor A . The GLOBOCAN 2022 cancer estimates: Data sources, methods, and a snapshot of the cancer burden worldwide. Int J Cancer. (2025) 156:1336–46. doi: 10.1002/ijc.35278 39688499

[3] Fan T Zhang M Yang J Zhu Z Cao W Dong C . Therapeutic cancer vaccines: advancements, challenges, and prospects. Signal Transd Tar. (2023) 8:450. doi: 10.1038/s41392-023-01674-3 38086815 PMC10716479

[4] Saxena M van der Burg SH Melief CJM Bhardwaj N . Therapeutic cancer vaccines. Nat Rev Cancer. (2021) 21:360–78. doi: 10.1038/s41568-021-00346-0 33907315

[5] Haanen JB Schumacher TNM Kagan JC Fritsch EF Wu CJ . Clinical development of cancer vaccines. Nat Med. (2026) 32:828–40. doi: 10.1038/s41591-026-04241-9 41814007

[6] Adamik J Munson PV Hartmann FJ Combes AJ Pierre P Krummel MF . Distinct metabolic states guide maturation of inflammatory and tolerogenic dendritic cells. Nat Commun. (2022) 13:5184. doi: 10.1038/s41467-022-32849-1 36056019 PMC9440236

[7] Palucka K Banchereau J . Cancer immunotherapy via dendritic cells. Nat Rev Cancer. (2012) 12:265–77. doi: 10.1038/nrc3258 22437871 PMC3433802

[8] Sheykhhasan M Ahmadieh-Yazdi A Heidari R Chamanara M Akbari M Poondla N . Revolutionizing cancer treatment: The power of dendritic cell-based vaccines in immunotherapy. BioMed Pharmacother. (2025) 184:117858. doi: 10.1016/j.biopha.2025.117858 39955851

[9] Tai Y Chen M Wang F Fan Y Zhang J Cai B . The role of dendritic cells in cancer immunity and therapeutic strategies. Int Immunopharmacol. (2024) 128:111548. doi: 10.1016/j.intimp.2024.111548 38244518

[10] Gardner TA Elzey BD Hahn NM . Sipuleucel-T (Provenge) autologous vaccine approved for treatment of men with asymptomatic or minimally symptomatic castrate-resistant metastatic prostate cancer. Hum Vacc Immunother. (2012) 8:534–9. doi: 10.4161/hv.19795 22832254

[11] Eisenbarth SC . Dendritic cell subsets in T cell programming: location dictates function. Nat Rev Immunol. (2019) 19:89–103. doi: 10.1038/s41577-018-0088-1 30464294 PMC7755085

[12] Pittet MJ Di Pilato M Garris C Mempel TR . Dendritic cells as shepherds of T cell immunity in cancer. Immunity. (2023) 56:2218–30. doi: 10.1016/j.immuni.2023.08.014 37708889 PMC10591862

[13] Moussion C Delamarre L . Antigen cross-presentation by dendritic cells: A critical axis in cancer immunotherapy. Semin Immunol. (2024) 71:101848. doi: 10.1016/j.smim.2023.101848 38035643

[14] Wang Y Xiang Y Xin VW Wang X-W Peng X-C Liu X-Q . Dendritic cell biology and its role in tumor immunotherapy. J Hematol Oncol. (2020) 13:107. doi: 10.1186/s13045-020-00939-6 32746880 PMC7397618

[15] Isnard S Hatton E Guillerme JB Hosmalin A . Monitoring antigen cross-presentation by human dendritic cells purified from peripheral blood. Method Enzymol. (2020) 635:283–305. doi: 10.1016/bs.mie.2020.01.004 32122551

[16] Satpathy AT Wu X Albring JC Murphy KM . Re(de)fining the dendritic cell lineage. Nat Immunol. (2012) 13:1145–54. doi: 10.1038/ni.2467 23160217 PMC3644874

[17] Zhu S Niu C Chen J . Transcriptional divergence between cDC1s and cDC2s: an AP1-IRF composite element-dependent program. Cell Mol Immunol. (2021) 18:1618–9. doi: 10.1038/s41423-020-00628-x 33473189 PMC8245613

[18] Brown CC Gudjonson H Pritykin Y Deep D Lavallee VP Mendoza A . Transcriptional basis of mouse and human dendritic cell heterogeneity. Cell. (2019) 179:846–863.e824. doi: 10.1016/j.cell.2019.09.035 31668803 PMC6838684

[19] Cruz FM Chan A Rock KL . Pathways of MHC I cross-presentation of exogenous antigens. Semin Immunol. (2023) 66:101729. doi: 10.1016/j.smim.2023.101729 36804685 PMC10023513

[20] Theisen D Murphy K . The role of cDC1s *in vivo*: CD8 T cell priming through cross-presentation. F1000Res. (2017) 6:98. doi: 10.12688/f1000research.9997.1 28184299 PMC5288679

[21] Zhang S Chopin M Nutt SL . Type 1 conventional dendritic cells: ontogeny, function, and emerging roles in cancer immunotherapy. Trends Immunol. (2021) 42:1113–27. doi: 10.1016/j.it.2021.10.004 34728143

[22] Perez CR De Palma M . Engineering dendritic cell vaccines to improve cancer immunotherapy. Nat Commun. (2019) 10:5408. doi: 10.1038/s41467-019-13368-y 31776331 PMC6881351

[23] Saito Y Komori S Kotani T Murata Y Matozaki T . The role of type-2 conventional dendritic cells in the regulation of tumor immunity. Cancers. (2022) 14:1976. doi: 10.3390/cancers14081976 35454882 PMC9028336

[24] Del Prete A Salvi V Soriani A Laffranchi M Sozio F Bosisio D . Dendritic cell subsets in cancer immunity and tumor antigen sensing. Cell Mol Immunol. (2023) 20:432–47. doi: 10.1038/s41423-023-00990-6 36949244 PMC10203372

[25] Ferris ST Durai V Wu R Theisen DJ Ward JP Bern MD . cDC1 prime and are licensed by CD4+ T cells to induce anti-tumour immunity. Nature. (2020) 584:624–9. doi: 10.1038/s41586-020-2611-3 32788723 PMC7469755

[26] Lei X Khatri I de Wit T de Rink I Nieuwland M Kerkhoven R . CD4(+) helper T cells endow cDC1 with cancer-impeding functions in the human tumor micro-environment. Nat Commun. (2023) 14:217. doi: 10.1038/s41467-022-35615-5 36639382 PMC9839676

[27] Lei X de Groot DC Welters MJP de Wit T Schrama E van Eenennaam H . CD4(+) T cells produce IFN-I to license cDC1s for induction of cytotoxic T-cell activity in human tumors. Cell Mol Immunol. (2024) 21:374–92. doi: 10.1038/s41423-024-01133-1 38383773 PMC10978876

[28] Harari A Graciotti M Bassani-Sternberg M Kandalaft LE . Antitumour dendritic cell vaccination in a priming and boosting approach. Nat Rev Drug Discov. (2020) 19:635–52. doi: 10.1038/s41573-020-0074-8 32764681

[29] Kuhn S Yang J Ronchese F . Monocyte-derived dendritic cells are essential for CD8(+) T cell activation and antitumor responses after local immunotherapy. Front Immunol. (2015) 6:584. doi: 10.3389/fimmu.2015.00584 26635798 PMC4655312

[30] Sun W Dai L Cao Y Pan P Zhi L Wang X . Monocytes reprogrammed by tumor microparticle vaccine inhibit tumorigenesis and tumor development. Cancer Nanotechnol. (2023) 14:34. doi: 10.1186/s12645-023-00190-x 37089435 PMC10106871

[31] Huang Q Wu X Wang Z Chen X Wang L Lu Y . The primordial differentiation of tumor-specific memory CD8(+) T cells as bona fide responders to PD-1/PD-L1 blockade in draining lymph nodes. Cell. (2022) 185:4049–4066.e4025. doi: 10.1016/j.cell.2022.09.020 36208623

[32] Laoui D Keirsse J Morias Y Van Overmeire E Geeraerts X Elkrim Y . The tumour microenvironment harbours ontogenically distinct dendritic cell populations with opposing effects on tumour immunity. Nat Commun. (2016) 7:13720. doi: 10.1038/ncomms13720 28008905 PMC5196231

[33] Shinde P Fernandes S Melinkeri S Kale V Limaye L . Compromised functionality of monocyte-derived dendritic cells in multiple myeloma patients may limit their use in cancer immunotherapy. Sci Rep-Uk. (2018) 8:5705. doi: 10.1038/s41598-018-23943-w 29632307 PMC5890285

[34] Grzes KM Sanin DE Kabat AM Stanczak MA Edwards-Hicks J Matsushita M . Plasmacytoid dendritic cell activation is dependent on coordinated expression of distinct amino acid transporters. Immunity. (2021) 54:2514–2530.e2517. doi: 10.1016/j.immuni.2021.10.009 34717796

[35] Cabeza-Cabrerizo M Cardoso A Minutti CM Pereira da Costa M Reis e Sousa C . Dendritic cells revisited. Annu Rev Immunol. (2021) 39:131–66. doi: 10.1146/annurev-immunol-061020-053707 33481643

[36] Murphy TL Murphy KM . Dendritic cells in cancer immunology. Cell Mol Immunol. (2022) 19:3–13. doi: 10.1038/s41423-021-00741-5 34480145 PMC8752832

[37] Anderson DA Dutertre CA Ginhoux F Murphy KM . Genetic models of human and mouse dendritic cell development and function. Nat Rev Immunol. (2021) 21:101–15. doi: 10.1038/s41577-020-00413-x 32908299 PMC10955724

[38] Marciscano AE Anandasabapathy N . The role of dendritic cells in cancer and anti-tumor immunity. Semin Immunol. (2021) 52:101481. doi: 10.1016/j.smim.2021.101481 34023170 PMC8545750

[39] Liu X Zhu R Luo Y Wang S Zhao Y Qiu Z . Distinct human Langerhans cell subsets orchestrate reciprocal functions and require different developmental regulation. Immunity. (2021) 54:2305–2320.e2311. doi: 10.1016/j.immuni.2021.08.012 34508661

[40] Raquer-McKay HM Maqueda-Alfaro RA Saravanan S Arroyo Hornero R Clausen BE Gottfried-Blackmore A . Monocytes give rise to Langerhans cells that preferentially migrate to lymph nodes at steady state. P Natl Acad Sci USA. (2024) 121:e2404927121. doi: 10.1073/pnas.2404927121 39541348 PMC11588065

[41] Wang J Parajuli N Wang Q Khalasawi N Peng H Zhang J . MiR-23a regulates skin Langerhans cell phagocytosis and inflammation-induced Langerhans cell repopulation. Biology-Basel. (2023) 12:925. doi: 10.3390/biology12070925 37508356 PMC10376168

[42] Sarivalasis A Boudousquie C Balint K Stevenson BJ Gannon PO Iancu EM . A phase I/II trial comparing autologous dendritic cell vaccine pulsed either with personalized peptides (PEP-DC) or with tumor lysate (OC-DC) in patients with advanced high-grade ovarian serous carcinoma. J Transl Med. (2019) 17:391. doi: 10.1186/s12967-019-02133-w 31771601 PMC6880492

[43] Li Z Feiyue Z Luyu H Wenqiang Z Ismail M Rueckert J-C . Neoantigen-based dendritic cell vaccines in lung cancer: overcoming immunosuppressive barriers for durable antitumor immunity. Cancer Treat Rev. (2026) 145:103125. doi: 10.1016/j.ctrv.2026.103125 41921304

[44] Luo S Chen J Xu F Chen H Li Y Li W . Dendritic cell-derived exosomes in cancer immunotherapy. Pharmaceutics. (2023) 15:2070. doi: 10.3390/pharmaceutics15082070 37631284 PMC10457773

[45] Zhang S Mo S Huang W Zhong D Yang X Xie S . Dendritic cell vaccines: Current research progress, challenges, and opportunities. Genes Dis. (2026) 13:101913. doi: 10.1016/j.gendis.2025.101913 41938709 PMC13050070

[46] Hotchkiss KM Batich KA Mohan A Rahman R Piantadosi S Khasraw M . Dendritic cell vaccine trials in gliomas: untangling the lines. Neuro-Oncology. (2023) 25:1752–62. doi: 10.1093/neuonc/noad088 37289203 PMC10547519

[47] Diao L Liu M . Rethinking antigen source: Cancer vaccines based on whole tumor cell/tissue lysate or whole tumor cell. Adv Sci. (2023) 10:2300121. doi: 10.1002/advs.202300121 37254712 PMC10401146

[48] Li Y Abudureheman A Xu J . Current progress in neoantigen-based dendritic cell vaccines for solid tumors. Cancer Biol Med. (2025) 22:1143–57. doi: 10.20892/j.issn.2095-3941.2025.0267 41024613 PMC12533755

[49] Dhar R Sonar S Das A Tajul Akmal NAS Hawa Jasni A RMT Balasubramaniam V . Dendritic cell-derived exosomes: Next generation of cancer immunotherapy. Biomedicines. (2025) 13:2497. doi: 10.3390/biomedicines13102497 41153781 PMC12561574

[50] Schioppa T Gaudenzi C Zucchi G Piserà A Vahidi Y Tiberio L . Extracellular vesicles at the crossroad between cancer progression and immunotherapy: focus on dendritic cells. J Transl Med. (2024) 22:691. doi: 10.1186/s12967-024-05457-4 39075551 PMC11288070

[51] Huang FY Dai SZ Wang JY Lin YY Wang CC Zheng WP . Engineered porous/hollow Burkholderia pseudomallei loading tumor lysate as a vaccine. Biomaterials. (2021) 278:121141. doi: 10.1016/j.biomaterials.2021.121141 34564035

[52] Liu J Fu M Wang M Wan D Wei Y Wei X . Cancer vaccines as promising immuno-therapeutics: platforms and current progress. J Hematol Oncol. (2022) 15:28. doi: 10.1186/s13045-022-01247-x 35303904 PMC8931585

[53] Li Q Zeng H Liu T Wang P Zhang R Zhao B . A dendritic cell vaccine for both vaccination and neoantigen-reactive T cell preparation for cancer immunotherapy in mice. Nat Commun. (2024) 15:10419. doi: 10.1038/s41467-024-54650-y 39613742 PMC11607313

[54] Cai Z Su X Qiu L Li Z Li X Dong X . Personalized neoantigen vaccine prevents postoperative recurrence in hepatocellular carcinoma patients with vascular invasion. Mol Cancer. (2021) 20:164. doi: 10.1186/s12943-021-01467-8 34903219 PMC8667400

[55] Xie N Shen G Huang C Zhu H . Neoantigen-driven personalized tumor therapy: An update from discovery to clinical application. Chin Med J-Peking. (2025) 138:2057–90. doi: 10.1097/CM9.0000000000003708 40757404 PMC12407177

[56] Li M Wang Y Wu P Zhang S Gong Z Liao Q . Application prospect of circular RNA-based neoantigen vaccine in tumor immunotherapy. Cancer Lett. (2023) 563:216190. doi: 10.1016/j.canlet.2023.216190 37062328

[57] Peng M Mo Y Wang Y Wu P Zhang Y Xiong F . Neoantigen vaccine: an emerging tumor immunotherapy. Mol Cancer. (2019) 18:128. doi: 10.1186/s12943-019-1055-6 31443694 PMC6708248

[58] Zhang R Yuan F Shu Y Tian Y Zhou B Yi L . Personalized neoantigen-pulsed dendritic cell vaccines show superior immunogenicity to neoantigen-adjuvant vaccines in mouse tumor models. Cancer Immunol Immun. (2020) 69:135–45. doi: 10.1007/s00262-019-02448-z 31807878 PMC6949210

[59] Pan X Ni S Hu K . Nanomedicines for reversing immunosuppressive microenvironment of hepatocellular carcinoma. Biomaterials. (2024) 306:122481. doi: 10.1016/j.biomaterials.2024.122481 38286109

[60] Kelley RK Sangro B Harris W Ikeda M Okusaka T Kang Y-K . Safety, efficacy, and pharmacodynamics of Tremelimumab plus Durvalumab for patients with unresectable hepatocellular carcinoma: randomized expansion of a phase I/II study. J Clin Oncol. (2021) 39:2991–3001. doi: 10.1200/JCO.20.03555 34292792 PMC8445563

[61] Vogel A Martinelli E . Updated treatment recommendations for hepatocellular carcinoma (HCC) from the ESMO Clinical Practice Guidelines. Ann Oncol. (2021) 32:801–5. doi: 10.1016/j.annonc.2021.02.014 33716105

[62] Wang Y Zhao Q Zhao B Zheng Y Zhuang Q Liao N . Remodeling tumor-associated neutrophils to enhance dendritic cell-based HCC neoantigen nano-vaccine efficiency. Adv Sci. (2022) 9:e2105631. doi: 10.1002/advs.202105631 35142445 PMC9009112

[63] Zhang W Guan J Wang W Chen G Fan L Lu Z . Neoantigen-specific mRNA/DC vaccines for effective anticancer immunotherapy. Genes Immun. (2024) 25:514–24. doi: 10.1038/s41435-024-00305-3 39592852

[64] Yang C Xue Y Duan Y Mao C Wan M . Extracellular vesicles and their engineering strategies, delivery systems, and biomedical applications. J Ctrl Rel. (2024) 365:1089–123. doi: 10.1016/j.jconrel.2023.11.057 38065416

[65] Maacha S Bhat AA Jimenez L Raza A Haris M Uddin S . Extracellular vesicles-mediated intercellular communication: roles in the tumor microenvironment and anti-cancer drug resistance. Mol Cancer. (2019) 18:55. doi: 10.1186/s12943-019-0965-7 30925923 PMC6441157

[66] van Niel G DAngelo G Raposo G . Shedding light on the cell biology of extracellular vesicles. Nat Rev Mol Cell Bio. (2018) 19:213–28. doi: 10.1038/nrm.2017.125 29339798

[67] Zhang X Zhang H Gu J Zhang J Shi H Qian H . Engineered extracellular vesicles for cancer therapy. Adv Mater. (2021) 33:2005709. doi: 10.1002/adma.202005709 33644908

[68] Marar C Starich B Wirtz D . Extracellular vesicles in immunomodulation and tumor progression. Nat Immunol. (2021) 22:560–70. doi: 10.1038/s41590-021-00899-0 33753940 PMC9389600

[69] Liu J Ren L Li S Li W Zheng X Yang Y . The biology, function, and applications of exosomes in cancer. Acta Pharm Sin B. (2021) 11:2783–97. doi: 10.1016/j.apsb.2021.01.001 34589397 PMC8463268

[70] Zhang X Cui H Zhang W Li Z Gao J . Engineered tumor cell-derived vaccines against cancer: The art of combating poison with poison. Bioact Mater. (2023) 22:491–517. doi: 10.1016/j.bioactmat.2022.10.016 36330160 PMC9619151

[71] Shamshiripour P Rahnama M Nikoobakht M Rad VF Moradi A-R Ahmadvand D . Extracellular vesicles derived from dendritic cells loaded with VEGF-A siRNA and doxorubicin reduce glioma angiogenesis *in vitro*. J Ctrl Rel. (2024) 369:128–45. doi: 10.1016/j.jconrel.2024.03.042 38522817

[72] Zhang H Lin W Kannan K Luo L Li J Chao PW . Aberrant chimeric RNA GOLM1-MAK10 encoding a secreted fusion protein as a molecular signature for human esophageal squamous cell carcinoma. Oncotarget. (2013) 4:2135–43. doi: 10.18632/oncotarget.1465 24243830 PMC3875775

[73] Xiong X Ke X Wang L Lin Y Wang S Yao Z . Neoantigen-based cancer vaccination using chimeric RNA-loaded dendritic cell-derived extracellular vesicles. J Extracell Vesicles. (2022) 11:e12243. doi: 10.1002/jev2.12243 35927827 PMC9451527

[74] Xu F Jiang D Xu J Dai H Fan Q Fei Z . Engineering of dendritic cell bispecific extracellular vesicles for tumor-targeting immunotherapy. Cell Rep. (2023) 42:113138. doi: 10.1016/j.celrep.2023.113138 37738123

[75] Shang L Jiang X Zhao X Huang X Wang X Jiang X . Mitochondrial DNA-boosted dendritic cell-based nanovaccination triggers antitumor immunity in lung and pancreatic cancers. Cell Rep Med. (2024) 5:101648. doi: 10.1016/j.xcrm.2024.101648 38986624 PMC11293323

[76] Wang F Ma S Liu Z Feng M Song D Li M . Dendritic cell-derived exosomes from PDT-treated glioma cells enhance antitumor immunity as a subcellular vaccine. J Transl Med. (2026) 24:703. doi: 10.1186/s12967-026-08111-3 41981674 PMC13214114

[77] Barbuto JA Ensina LF Neves AR Bergami-Santos P Leite KR Marques R . Dendritic cell-tumor cell hybrid vaccination for metastatic cancer. Cancer Immunol Immun. (2004) 53:1111–8. doi: 10.1007/s00262-004-0551-7 15185011 PMC11032787

[78] Kim JH Lee Y Bae YS Kim WS Kim K Im HY . Phase I/II study of immunotherapy using autologous tumor lysate-pulsed dendritic cells in patients with metastatic renal cell carcinoma. Clin Immunol. (2007) 125:257–67. doi: 10.1016/j.clim.2007.07.014 17916447

[79] Wheeler CJ Black KL . DCVax-Brain and DC vaccines in the treatment of GBM. Expert Opin Inv Drug. (2009) 18:509–19. doi: 10.1517/13543780902841951 19335279

[80] McKay RR Hafron JM Ferro C Wilfehrt HM Fitch K Flanders SC . A retrospective observational analysis of overall survival with Sipuleucel-T in Medicare beneficiaries treated for advanced prostate cancer. Adv Ther. (2020) 37:4910–29. doi: 10.1007/s12325-020-01509-5 33029725 PMC7596004

[81] Kumar C Kohli S Chiliveru S Bapsy PP Jain M Suresh Attili VS . A retrospective analysis comparing APCEDEN(®) dendritic cell immunotherapy with best supportive care in refractory cancer. Immunotherapy-Uk. (2017) 9:889–97. doi: 10.2217/imt-2017-0064 28838282

[82] Rosenquist R Bernard E Erkers T Scott DW Itzykson R Rousselot P . Novel precision medicine approaches and treatment strategies in hematological malignancies. J Intern Med. (2023) 294:413–36. doi: 10.1111/joim.13697 37424223

[83] Zhao A Zhou H Yang J Li M Niu T . Epigenetic regulation in hematopoiesis and its implications in the targeted therapy of hematologic malignancies. Signal Transd Tar. (2023) 8:71. doi: 10.1038/s41392-023-01342-6 36797244 PMC9935927

[84] Kitawaki T . DC-based immunotherapy for hematological malignancies. Int J Hematol. (2014) 99:117–22. doi: 10.1007/s12185-013-1496-4 24379028

[85] Floisand Y Remberger M Bigalke I Josefsen D Valerhaugen H Inderberg EM . WT1 and PRAME RNA-loaded dendritic cell vaccine as maintenance therapy in de novo AML after intensive induction chemotherapy. Leukemia. (2023) 37:1842–9. doi: 10.1038/s41375-023-01980-3 37507426

[86] Chung DJ Sharma S Rangesa M DeWolf S Elhanati Y Perica K . Langerhans dendritic cell vaccine bearing mRNA-encoded tumor antigens induces antimyeloma immunity after autotransplant. Blood Adv. (2022) 6:1547–58. doi: 10.1182/bloodadvances.2021005941 35100339 PMC8905697

[87] Tu S Zhou L Huang R Zhou X Yang J He Y . Dendritic cell vaccines extend CAR T-cell persistence and improve the efficacy of CD19 CAR T-cell therapy in refractory or relapsed adult B-ALL patients. Am J Hematol. (2024) 99:1437–40. doi: 10.1002/ajh.27349 38712616

[88] Li C Wang D Hasegawa A Liu H Zhang P Bao Y . Dendritic cell–based vaccines (KSD-101) against EBV-associated lymphoproliferative diseases: Results from an ongoing phase I clinical study. J Clin Oncol. (2025) 43:e14515–5. doi: 10.1200/JCO.2025.43.16_suppl.e14515 42148471

[89] Pouyan A Ghorbanlo M Eslami M Jahanshahi M Ziaei E Salami A . Glioblastoma multiforme: insights into pathogenesis, key signaling pathways, and therapeutic strategies. Mol Cancer. (2025) 24:58. doi: 10.1186/s12943-025-02267-0 40011944 PMC11863469

[90] Liau LM Ashkan K Brem S Campian JL Trusheim JE Iwamoto FM . Association of autologous tumor lysate-loaded dendritic cell vaccination with extension of survival among patients with newly diagnosed and recurrent glioblastoma: a phase 3 prospective externally controlled cohort trial. JAMA Oncol. (2023) 9:112–21. doi: 10.1001/jamaoncol.2022.5370 36394838 PMC9673026

[91] Bota DA Taylor TH Piccioni DE Duma CM LaRocca RV Kesari S . Phase 2 study of AV-GBM-1 (a tumor-initiating cell targeted dendritic cell vaccine) in newly diagnosed Glioblastoma patients: safety and efficacy assessment. J Exp Clin Canc Res. (2022) 41:344. doi: 10.1186/s13046-022-02552-6 36517865 PMC9749349

[92] Georges J Clay CM Amin S Ifrach J Burns BA Trivedi A . Vaccination by homologous antigenic loading with DOC1021 as adjuvant therapy for glioblastoma: phase I clinical trial results. J Clin Oncol. (2025) 43:2014. doi: 10.1200/JCO.2025.43.16_suppl.2014

[93] Ding Z Li Q Zhang R Xie L Shu Y Gao S . Personalized neoantigen pulsed dendritic cell vaccine for advanced lung cancer. Signal Transd Tar. (2021) 6:26. doi: 10.1038/s41392-020-00448-5 33473101 PMC7817684

[94] Ingels J De Cock L Stevens D Mayer RL Thery F Sanchez GS . Neoantigen-targeted dendritic cell vaccination in lung cancer patients induces long-lived T cells exhibiting the full differentiation spectrum. Cell Rep Med. (2024) 5:101516. doi: 10.1016/j.xcrm.2024.101516 38626769 PMC11148567

[95] Koido S Taguchi J Shimabuku M Kan S Bito T Misawa T . Dendritic cells pulsed with multifunctional Wilms’ tumor 1 (WT1) peptides combined with multiagent chemotherapy modulate the tumor microenvironment and enable conversion surgery in pancreatic cancer. J Immunother Cancer. (2024) 12:e009765. doi: 10.1136/jitc-2024-009765 39384197 PMC11474828

[96] Land FRVT Willemsen M Bezemer K Burg SHVD Bosch TPPVD Doukas M . Dendritic cell–based immunotherapy in patients with resected pancreatic cancer. J Clin Oncol. (2024) 42:3083–93. doi: 10.1200/jco.23.02585 38950309 PMC11379361

[97] Sun B Ding P Song Y Zhou J Chen X Peng C . FDX1 downregulation activates mitophagy and the PI3K/AKT signaling pathway to promote hepatocellular carcinoma progression by inducing ROS production. Redox Biol. (2024) 75:103302. doi: 10.1016/j.redox.2024.103302 39128228 PMC11366913

[98] Liang P Yu J Wang L Chen S-T Dong G Hou Q-D . A first-in-human study to evaluate a personalized neoantigen-based mRNA-loaded dendritic cell vaccine, in combination with ablation, in patients with hepatocellular carcinoma. J Clin Oncol. (2024) 42:e14513–3. doi: 10.1200/JCO.2024.42.16_suppl.e14513 42148471

[99] Ge Y Xi H Ju S Zhang X . Blockade of PD-1/PD-L1 immune checkpoint during DC vaccination induces potent protective immunity against breast cancer in hu-SCID mice. Cancer Lett. (2013) 336:253–9. doi: 10.1016/j.canlet.2013.03.010 23523609

[100] Rosenblatt J Glotzbecker B Mills H Vasir B Tzachanis D Levine JD . PD-1 blockade by CT-011, anti-PD-1 antibody, enhances ex vivo T-cell responses to autologous dendritic cell/myeloma fusion vaccine. J Immunother. (2011) 34:409–18. doi: 10.1097/CJI.0b013e31821ca6ce 21577144 PMC3142955

[101] Gupta YH Khanom A Acton SE . Control of dendritic cell function within the tumour microenvironment. Front Immunol. (2022) 13. doi: 10.3389/fimmu.2022.733800 35355992 PMC8960065

[102] Moon CY Belabed M Park MD Mattiuz R Puleston D Merad M . Dendritic cell maturation in cancer. Nat Rev Cancer. (2025) 25:225–48. doi: 10.1038/s41568-024-00787-3 39920276 PMC11954679

[103] Xiao Z Wang J Yang J Guo F Zhang L Zhang L . Dendritic cells instruct T cell anti-tumor immunity and immunotherapy response. Innovation Med. (2025) 3:100128. doi: 10.59717/j.xinn-med.2025.100128

[104] Liu T Zhao J Yao Z Zhou J Gong H Jin Z . Recent advances in natural products for cancer immunotherapy. Phytother Res. (2026) 40:1661–95. doi: 10.1002/ptr.70218 41605604

[105] Kaczmarek M Poznanska J Fechner F Michalska N Paszkowska S Napierała A . Cancer vaccine therapeutics: Limitations and effectiveness-a literature review. Cells. (2023) 12:2159. doi: 10.3390/cells12172159 37681891 PMC10486481

[106] Roy S Sethi TK Taylor D Kim YJ Johnson DB . Breakthrough concepts in immune-oncology: Cancer vaccines at the bedside. J Leukocyte Biol. (2020) 108:1455–89. doi: 10.1002/jlb.5bt0420-585rr 32557857

[107] Meskini M Amanzadeh A Salehi F Bouzari S Karimipoor M Fuso A . A protocol to isolate and characterize pure monocytes and generate monocyte-derived dendritic cells through FBS-coated flasks. Sci Rep-Uk. (2024) 14:23956. doi: 10.1038/s41598-024-75376-3 39397067 PMC11471755

[108] Si F Liu X Tao Y Zhang Y Ma F Hsueh EC . Blocking senescence and tolerogenic function of dendritic cells induced by γδ Treg cells enhances tumor-specific immunity for cancer immunotherapy. J Immunother Cancer. (2024) 12:e008219. doi: 10.1136/jitc-2023-008219 38580332 PMC11002396

[109] Jing H Gao Y Sun Z Liu S . Recent advances in novel tumor immunotherapy strategies based on regulating the tumor microenvironment and immune checkpoints. Front Immunol. (2025) 16. doi: 10.3389/fimmu.2025.1529403 40607438 PMC12213813

[110] Radojevic D Tomic S Mihajlovic D Tolinacki M Pavlovic B Vucevic D . Fecal microbiota composition associates with the capacity of human peripheral blood monocytes to differentiate into immunogenic dendritic cells *in vitro*. Gut Microbes. (2021) 13:1921927. doi: 10.1080/19490976.2021.1921927 33970783 PMC8115579

[111] Chen W Li Z Tang J Liu S . Dendritic cell-based immunotherapy for head and neck squamous cell carcinoma: Advances and challenges. Front Immunol. (2025) 16:1573635. doi: 10.3389/fimmu.2025.1573635 40491907 PMC12146400

[112] Pearlman AH Hwang MS Konig MF Hsiue EH Douglass J DiNapoli SR . Targeting public neoantigens for cancer immunotherapy. Nat Cancer. (2021) 2:487–97. doi: 10.1038/s43018-021-00210-y 34676374 PMC8525885

[113] Leong SL Murdolo L Maddumage JC Koutsakos M Kedzierska K Purcell AW . Characterisation of novel influenza-derived HLA-B*18:01-restricted epitopes. Clin Transl Immunol. (2024) 13:e1509. doi: 10.1002/cti2.1509 38737448 PMC11087170

[114] Wang H Yang X Hu C Huang C Wang H Zhu D . Programmed polymersomes with spatio-temporal delivery of antigen and dual-adjuvants for efficient dendritic cells-based cancer immunotherapy. Chin Chem Lett. (2022) 33:4179–84. doi: 10.1016/j.cclet.2022.02.022 38826717

[115] Granato AM Pancisi E Piccinini C Stefanelli M Pignatta S Soldati V . Dendritic cell vaccines as cancer treatment: Focus on 13 years of manufacturing and quality control experience in advanced therapy medicinal products. Cytotherapy. (2024) 26:1547–55. doi: 10.1016/j.jcyt.2024.07.005 39046388

[116] Katsikis PD Ishii KJ Schliehe C . Challenges in developing personalized neoantigen cancer vaccines. Nat Rev Immunol. (2024) 24:213–27. doi: 10.1038/s41577-023-00937-y 37783860 PMC12001822

[117] Boudousquie C Boand V Lingre E Dutoit L Balint K Danilo M . Development and optimization of a GMP-compliant manufacturing process for a personalized tumor lysate dendritic cell vaccine. Vaccines-Basel. (2020) 8:25. doi: 10.3390/vaccines8010025 31947581 PMC7157441

[118] Sun Q Welsh KJ Bruns DE Sacks DB Zhao Z . Inadequate reporting of analytical characteristics of biomarkers used in clinical research: A threat to interpretation and replication of study findings. Clin Chem. (2019) 65:1554–62. doi: 10.1373/clinchem.2019.309575 31672858 PMC7055667

[119] Shemesh CS Hsu JC Hosseini I Shen BQ Rotte A Twomey P . Personalized cancer vaccines: Clinical landscape, challenges, and opportunities. Mol Ther. (2021) 29:555–70. doi: 10.1016/j.ymthe.2020.09.038 33038322 PMC7854282

[120] Zafar A Khatoon S Khan MJ Abu J Naeem A . Advancements and limitations in traditional anti-cancer therapies: A comprehensive review of surgery, chemotherapy, radiation therapy, and hormonal therapy. Discov Oncol. (2025) 16:607. doi: 10.1007/s12672-025-02198-8 40272602 PMC12021777

[121] Heras-Murillo I Adan-Barrientos I Galan M Wculek SK Sancho D . Dendritic cells as orchestrators of anticancer immunity and immunotherapy. Nat Rev Clin Oncol. (2024) 21:257–77. doi: 10.1038/s41571-024-00859-1 38326563

[122] Rob L Cibula D Knapp P Mallmann P Klat J Minar L . Safety and efficacy of dendritic cell-based immunotherapy DCVAC/OvCa added to first-line chemotherapy (carboplatin plus paclitaxel) for epithelial ovarian cancer: A phase 2, open-label, multicenter, randomized trial. J Immunother Cancer. (2022) 10:e003190. doi: 10.1136/jitc-2021-003190 34992091 PMC8739446

[123] Ding J Zheng Y Wang G Zheng J Chai D . The performance and perspectives of dendritic cell vaccines modified by immune checkpoint inhibitors or stimulants. Bba-Rev Cancer. (2022) 1877:188763. doi: 10.1016/j.bbcan.2022.188763 35872287

[124] Brandenburg A Heine A Brossart P . Next-generation cancer vaccines and emerging immunotherapy combinations. Trends Cancer. (2024) 10:749–69. doi: 10.1016/j.trecan.2024.06.003 39048489

[125] Guo Z Yuan Y Chen C Lin J Ma Q Liu G . Durable complete response to neoantigen-loaded dendritic-cell vaccine following anti-PD-1 therapy in metastatic gastric cancer. NPJ Precis Oncol. (2022) 6:34. doi: 10.1038/s41698-022-00279-3 35661819 PMC9166775

[126] Liang L Wen Y Hu R Wang L Xia Y Hu C . Safety and efficacy of PD-1 blockade-activated multiple antigen-specific cellular therapy alone or in combination with apatinib in patients with advanced solid tumors: A pooled analysis of two prospective trials. Cancer Immunol Immun. (2019) 68:1467–77. doi: 10.1007/s00262-019-02375-z 31451841 PMC11028216

[127] Han Y Wu Y Yang C Huang J Guo Y Liu L . Dynamic and specific immune responses against multiple tumor antigens were elicited in patients with hepatocellular carcinoma after cell-based immunotherapy. J Transl Med. (2017) 15:64. doi: 10.1186/s12967-017-1165-0 28330473 PMC5363021

[128] Zhou Y Li M Zhang B Yang C Wang Y Zheng S . A pilot study of multi-antigen stimulated cell therapy-I plus camrelizumab and apatinib in patients with advanced bone and soft-tissue sarcomas. BMC Med. (2023) 21:470. doi: 10.1186/s12916-023-03132-x 38031088 PMC10687909

[129] Shi Y Chen M An X Xue C Shu D Li H . Updated results on multiple antigens stimulating cellular therapy (MASCT-I) in metastatic urothelial carcinoma: A randomized, open-label, phase I trial. J Clin Oncol. (2024) 42:2545. doi: 10.1200/JCO.2024.42.16_suppl.2545 42148471

[130] Chen Y Zheng Y Shi H Yang W Xu H Li Y . Personalized neoantigen DC vaccine combined with camrelizumab following definitive therapy in locally advanced unresectable esophageal squamous cell carcinoma (CHANT-241): Protocol for a randomized controlled trial. Front Immunol. (2025) 16:1658670. doi: 10.3389/fimmu.2025.1658670 41601631 PMC12833302

[131] Vo MC Yang S Jung SH Chu TH Lee HJ Lakshmi TJ . Synergistic antimyeloma activity of dendritic cells and pomalidomide in a murine myeloma model. Front Immunol. (2018) 9:1798. doi: 10.3389/fimmu.2018.01798 30123221 PMC6085413

[132] Vo M-C Jung S-H Chu T-H Lee H-J Lakshmi TJ Park H-S . Lenalidomide and programmed death-1 blockade synergistically enhances the effects of dendritic cell vaccination in a model of murine myeloma. Front Immunol. (2018) 9. doi: 10.3389/fimmu.2018.01370 29967612 PMC6015916

[133] Abakushina EV Popova LI Zamyatnin AA Werner J Mikhailovsky NV Bazhin AV . The advantages and challenges of anticancer dendritic cell vaccines and NK cells in adoptive cell immunotherapy. Vaccines-Basel. (2021) 9:1363. doi: 10.3390/vaccines9111363 34835294 PMC8625865

[134] Liu S Galat V Galat Y Lee YKA Wainwright D Wu J . NK cell-based cancer immunotherapy: From basic biology to clinical development. J Hematol Oncol. (2021) 14:7. doi: 10.1186/s13045-020-01014-w 33407739 PMC7788999

[135] Myers JA Miller JS . Exploring the NK cell platform for cancer immunotherapy. Nat Rev Clin Oncol. (2021) 18:85–100. doi: 10.1038/s41571-020-0426-7 32934330 PMC8316981

[136] Nagai H Karube R . Late-stage ovarian cancer with systemic multiple metastases shows marked shrinkage using a combination of Wilms' tumor antigen 1 (WT1) dendritic cell vaccine, natural killer (NK) cell therapy, and nivolumab. Cureus. (2024) 16:e56685. doi: 10.7759/cureus.56685 38523872 PMC10960621

[137] Chu T-H Vo M-C Lakshmi TJ Ahn S-Y Kim M Song G-Y . Novel IL-15 dendritic cells have a potent immunomodulatory effect in immunotherapy of multiple myeloma. Transl Oncol. (2022) 20:101413. doi: 10.1016/j.tranon.2022.101413 35413499 PMC9006865

[138] Liu T . Challenges in cardio-oncology and psycho-oncology. Heart Mind. (2025) 9:85–6. doi: 10.4103/hm.HM-D-25-00046

